# A Simple Method for Visualization of Locus-Specific H4K20me1 Modifications in Living *Caenorhabditis elegans* Single Cells

**DOI:** 10.1534/g3.118.200333

**Published:** 2018-05-03

**Authors:** Yoichi Shinkai, Masahiro Kuramochi, Motomichi Doi

**Affiliations:** Molecular Neurobiology Research Group and DAI-Lab, Biomedical Research Institute, National Institute of Advanced Industrial Science and Technology (AIST), Central 6, 1-1-1, Higashi, Tsukuba, Ibaraki, 305-8566, Japan

**Keywords:** *in vivo* visualization, single-gene resolution, histone modification, mintbody

## Abstract

Recently, advances in next-generation sequencing technologies have enabled genome-wide analyses of epigenetic modifications; however, it remains difficult to analyze the states of histone modifications at a single-cell resolution in living multicellular organisms because of the heterogeneity within cellular populations. Here we describe a simple method to visualize histone modifications on the specific sequence of target locus at a single-cell resolution in living *Caenorhabditis elegans*, by combining the LacO/LacI system and a genetically-encoded H4K20me1-specific probe, “mintbody”. We demonstrate that Venus-labeled mintbody and mTurquoise2-labeled LacI can co-localize on an artificial chromosome carrying both the target locus and LacO sequences, where H4K20me1 marks the target locus. We demonstrate that our visualization method can precisely detect H4K20me1 depositions on the *her-1* gene sequences on the artificial chromosome, to which the dosage compensation complex binds to regulate sex determination. The degree of H4K20me1 deposition on the *her-1* sequences on the artificial chromosome correlated strongly with sex, suggesting that, using the artificial chromosome, this method can reflect context-dependent changes of H4K20me1 on endogenous genomes. Furthermore, we demonstrate live imaging of H4K20me1 depositions on the artificial chromosome. Combined with ChIP assays, this mintbody-LacO/LacI visualization method will enable analysis of developmental and context-dependent alterations of locus-specific histone modifications in specific cells and elucidation of the underlying molecular mechanisms.

The wide variety of post-translational modifications of histone proteins has crucial roles in the regulation of gene expression and genomic integrity. Histone modification pattern profiles change in response to environmental and developmental stimuli and affect the functional properties of cells in various organisms (Heintzman *et al.* 2009). Recent studies have also reported aberrant changes of histone modifications in multiple human disorders (Portela and Esteller 2010; Kofink *et al.* 2013). Therefore, the roles of histone modifications, along with their real-time dynamics, are fundamental questions for epigenetic studies.

Real-time analyses of cell- or tissue-specific epigenetic states in live animals will facilitate precise understanding of the dynamical control of epigenetic modifications and how they affect cell and animal phenotypic traits. Although various techniques have been used to analyze histone modification states, detection of the live-cell dynamics of histone modification at single-locus resolution has proven difficult (Bheda and Schneider 2014). In particular, advances in next-generation sequencing technology have greatly contributed to our understanding of the genome-wide status of histone modifications; however, the heterogeneity of cellular populations in multicellular organisms and the requirement for cell lysis or fixation, have prevented single-cell and real-time assessment of histone modifications. In addition, although methods of visualizing global histone modification levels in single live cells have been reported (Hayashi-Takanaka *et al.* 2011; Ito *et al.* 2011), these techniques do not permit the analysis of locus-specific histone modifications.

Histone H4 Lys20 mono-methylation (H4K20me1) is an essential histone modification involved in gene regulation, DNA repair, cell cycle progression, and mitotic chromosome condensation (Beck *et al.* 2012). In addition, H4K20 methylation is involved in neuronal development, memory formation, and senescence (Wang *et al.* 2015; Tanaka *et al.* 2017; Delaney *et al.* 2017), suggesting that levels of H4K20 modification are likely dynamically altered during neuronal maturation and maintenance; however, the spatiotemporal dynamics of H4K20me1 modification during such phenomena remain to be elucidated.

Recently, a genetically encoded single-chain antibody fragment recognizing H4K20me1, referred to as mintbody, was developed to visualize the temporal dynamics of global histone modification (Sato *et al.* 2016). Here, we capitalize on the development of mintbody to detect locus-specific H4K20me1 enrichment in single cells of living worms. To examine H4K20me1 modification at a single locus in living *C. elegans*, we focused on methylation of the *her-1* gene, a regulator of sex determination. In *C. elegans*, the dosage compensation complex (DCC) binds to X chromosomes of hermaphrodite worms and is required for enrichment of H4K20me1 (Vielle *et al.* 2012; Wells *et al.* 2012). H4K20me1 contributes to global repression of X chromosomes (Kramer *et al.* 2015; Brejc *et al.* 2017). In addition to the X chromosome, DCC also directly binds to the *her-1* gene locus on autosome V in a sex-dependent manner, to regulate the transcription of *her-1* which is important for sex determination (Chu *et al.* 2002; Ercan *et al.* 2007). Interestingly, ectopic binding of DCC is sufficient for the deposition of H4K20me1 on other autosomes (Vielle *et al.* 2012; Brejc *et al.* 2017). Using the LacO/LacI system and artificial chromosomes (Gonzalez-Serricchio and Sternberg 2006), we show that the H4K20me1-mintbody allows simple visualization of the *her-1* sequence-dependent H4K20me1 in living worms (mintbody-LacO/LacI system). Moreover, the level of H4K20me1 deposition on the artificial chromosome correlated strongly with worm sex, suggesting that this method can reflect the H4K20me1 modification of endogenous genomes.

## Materials and Methods

### Strains and culture

Wild-type strain N2 and *him-5(e1490)* were used in this study. *him-5(e1490)* was used for the analysis of sex-dependent H4K20me1 deposition. All strains were cultured on Nematode Growth Medium (NGM) plates with *E. coli* strain OP-50 under standard conditions (Brenner 1974).

### DNA constructs and germline transformation

For expression of the H4K20me1-mintbody, we substituted Venus for the GFP of the original H4K20me1-mintbody (a gift from H. Kimura and Y. Sato) using In-Fusion technology (TaKaRa). The *unc-86* promoter was inserted into the multiple-cloning site of the H4K20me1-mintbody vector. For the mTurquoise2::LacI plasmid, the CFP region in the original LacI construct (a gift from S. Mango) was substituted with mTurquoise2, and the *htz-1* promoter was inserted upstream of the mTurquoise2::LacI sequence.

For construction of artificial chromosomes containing various target gene sequences, the *her-1* gene locus (3164 bp) including the *her-1* promoter region (2183 bp) and coding region from the first exon to the middle of the second intron (981 bp) was amplified by PCR from the N2 genome and subcloned into the pPD49.26 vector. The short *her-1* gene locus, (2683 bp) lacking one DCC binding site, was amplified by PCR and subcloned into the pPD49.26 vector. One DCC binding site in the second intron of *her-1* was mutated as previously reported (Chu *et al.* 2002) (WT: CAAAAACTGAGCCTG >> mutant: acagactgcagatac). The *abts-1.b* promoter (1.1 kb) was amplified by PCR from N2 genomic DNA. The *rex-1* sequence (ggctgcgggtaattgggcaggggaaagaagaat) was added to the 5′ end of the *abts-1.b* promoter by PCR using primers containing the *rex-1* sequence.

Transgenic strains were generated by microinjection of test DNA (10–50 ng/μL), along with co-injection marker DNA (*Pmyo-2*::*gfp*, *Pmyo-2*::*mCherry*, or *Punc-122*::*gfp*; 2–20 ng/μL), and carrier DNA (0–70 ng/μL), as described (Mello and Fire 1995). For expression of the H4K20me1-mintbody and mTurquoise2::LacI, PCR products encoding the *Punc-86*::H4K20me1-mintbody, *Phtz-1*::mTurquoise2::LacI, and the co-injection marker *Punc-122*::*gfp* were injected into wild-type worms. Transgenes harboring various gene sequences and LacO repeats were generated by injecting each plasmid DNA containing target gene sequence described above and pSV2-DHFR8.32, which contains 256 copies of the LacO sequence, as well as the co-injection marker *Pmyo-2*::*gfp* or *Pmyo-2*::*mCherry*. Transgenes carrying *rex-1*::*abts-1.b* and LacO sequences were integrated into the genome using a previously reported method (Mariol *et al.* 2013). The integrated array was inserted into a chromosome other than X, since XO males carrying the integrated array generated XO male progenies carrying the integrated array when mated with XX hermaphrodites.

### Analysis of co-localization

Images were acquired from live worms at L4 larval stage using an Olympus FV1000 confocal microscope equipped with a 60x objective lens. Image acquisition was done at a resolution of 640 × 640 pixels with 4× zoom. Worms were mounted on 5% agar pads containing 5 mM sodium azide. PLM and PVM neuronal cells were identified based on the YFP fluorescence generated by H4K20me1-mintbody expression driven by the *unc-86* promoter and its relative location. For analysis of co-localization, two-dimensional XY images were captured from a center of each nucleus of cells. All nuclei of PLM and PVM neuronal cells having the YFP fluorescence and LacI::mTurquoise2 spots were analyzed. For time-lapse imaging, worms were mounted on 5% agar pads containing 0.05 μm microsphere polystyrene beads (Polysciences, Inc., #08691) to prevent worms from moving during image acquisition. Images were acquired every 30 min for 5 hr at a resolution of 512 × 256 pixels with 5× zoom.

### Statistical analysis

Pearson’s correlation coefficient (PCC) values for H4K20me1-mintbody and mTurquoise2::LacI co-localization were determined for each nucleus, since both were localized to this organelle. Using the sparse distribution of the H4K20me1-mintbody throughout the entire nucleus as a background signal, auto-thresholding and subsequent binarization allowed identification of the edge of the nucleus. Then, we measured PCC values in the area encompassed by the edge of each nucleus and compared mean PCC values among different groups. A series of image processing and PCC measurement was performed using ImageJ with macro.

All data points were collected from more than 24 worms. For visual inspection, if the YFP (H4K20me1-mintbody) and CFP (mTurquoise2::LacI) signals appeared to have the same morphologies and locations, spots were considered co-localized. P values were calculated using the Fisher’s exact test; asterisks indicate statistically significant differences (*P < 0.01). For comparisons based on PCC values, p values were calculated using the Student’s *t*-test; asterisks indicate statistically significant differences (*P < 0.01).

### Data availability

Strains are available upon request. The authors state that all data necessary for confirming the conclusions presented in the article are represented fully within the article. Supplemental material available at Figshare: https://doi.org/10.25387/g3.6157781.

## Results and Discussion

**Figure 1:**
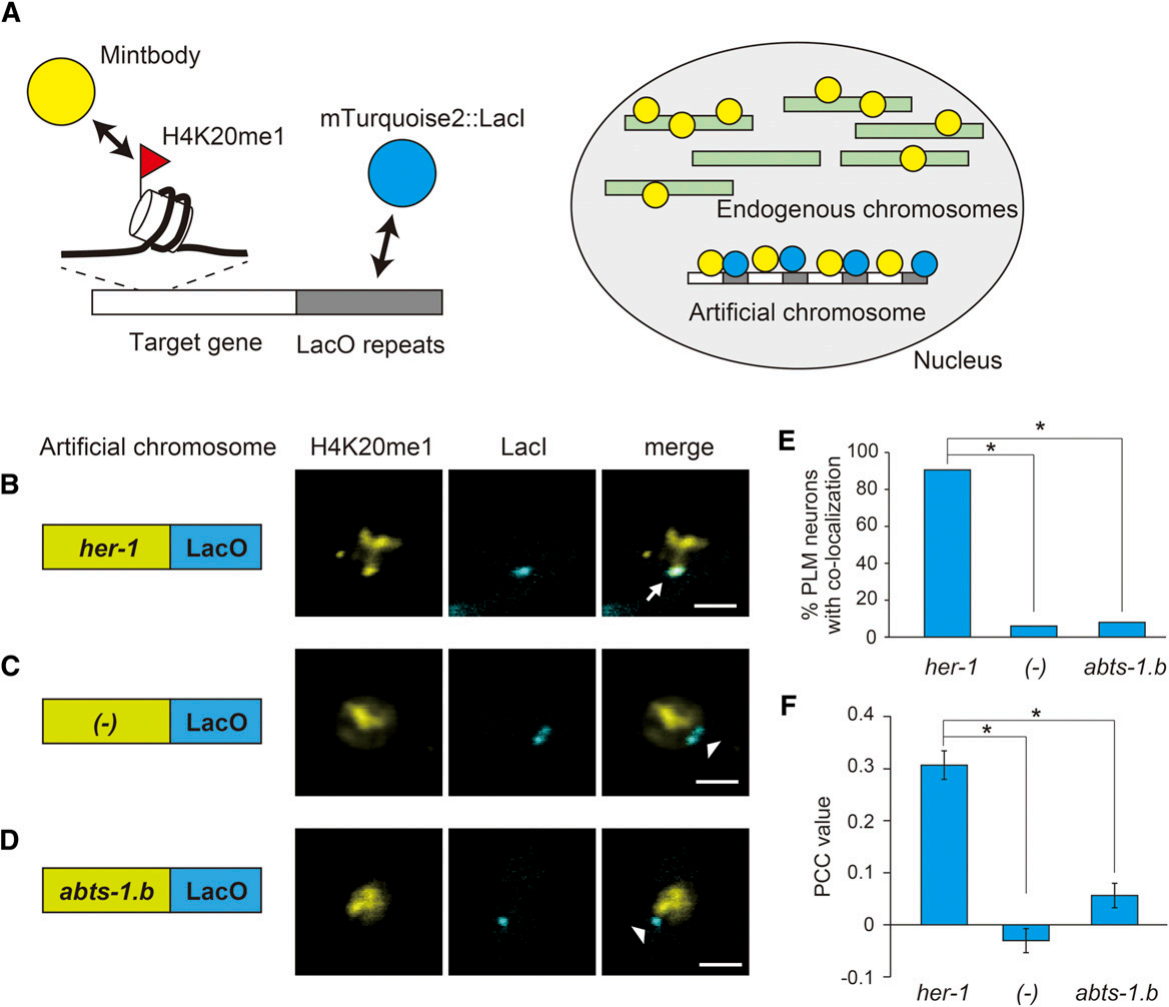
*In vivo* visualization of target gene-specific H4K20me1 in a single neuron using the mintbody-LacO/LacI system. (A) Schematic diagram of the mintbody-LacO/LacI system. White rectangles indicate target DNA sequence from a specific gene locus. Gray rectangles indicate LacO sequences. Green rectangles indicate endogenous chromosomes. Double-headed arrows indicate physical interaction. (B)–(D) Localization of H4K20me1-mintbody and the artificial chromosome carrying the *her-1* promoter and LacO sequences (B), only LacO sequences (C), or *abts-1.b* promoter and LacO sequences (D) in PLM neurons at the L4 larval stage. Artificial chromosomes were visualized by the binding of mTurquoise2::LacI. Arrows indicate the co-localization of H4K20me1-mintbody and the artificial chromosome. Arrowheads indicate exclusion of H4K20me1-mintbody from the artificial chromosome. White scale bars, 2 µm. (E) Quantitative evaluation of the co-localization of H4K20me1-mintbody and artificial chromosomes, corresponding to (B)–(D). *P < 0.01. (F) Mean PCC values of the co-localization of H4K20me1-mintbody and artificial chromosomes, corresponding to (E). *P < 0.01.

Visualization of histone modifications on a specific gene locus in a living organism requires several criteria to be fulfilled: i) the animals must be never fixed or lysed; ii) the histone modifications should be amplified to obtain sufficient signal from the small amount of the histone modification at a specific gene locus; and iii) the histone modification of interest must be distinguished from other histone modifications on the genome. To meet these criteria, we used “mintbody,” a single-chain antibody tagged with a fluorescent protein, the Lac repressor (LacI) tagged with another fluorescent protein, and artificial chromosomes carrying multiple copies of DNA fragments from a specific gene locus and a Lac operator sequence (LacO). The genetically encoded mintbody binds to a particular histone modification and enables genome-wide visualization of histone modifications in living organisms without cell fixation and lysis (Sato *et al.* 2013, 2016). Since the artificial chromosome system has been used extensively to analyze the expression pattern of genes of interest in *C. elegans*, the histone modifications amplified by multiple gene duplication on the artificial chromosome may reflect those on endogenous chromosomes, and can be distinguished from other histone modifications on the genome by the co-localization of mintbody and LacI (Figure 1A).

To examine whether histone modifications at specific gene sequences can be visualized in living animals, we first focused on histone H4 Lys20 mono-methylation of the *her-1* gene in neuronal cells, which should differ in a sex-dependent manner. For this purpose, we generated transgenic worms expressing a Venus-tagged mintbody that specifically bound to histone H4 Lys20 mono-methylation (H4K20me1-mintbody) and LacI fused with mTurquoise2 (mTurquoise2::LacI). Then, an artificial chromosome carrying multiple copies of LacO repeats and *her-1* gene sequences was introduced into the transgenic worms. Since expression of H4K20me1-mintbody was driven by *unc-86* promoter which is active in a subset of neurons, we then observed the nuclei of several neurons in the worms, and found that the mTurquoise2::LacI fluorescence spot overlapped with that generated by the H4K20me1-mintbody (Figure 1A, B, Figure S1, and Supplementary Movie 1). The overlap was observed in all neurons expressing these proteins such as PLM, HSN, PLN, PVM, PQR, and ALN cells. Besides neurons, similar co-localization of H4K20me1-mintbody and mTurquoise2 in the nucleus was observed in other cells such as the intestine (Figure S2). Since the X chromosome undergoes chromosome-wide H4K20 mono-methylation (Kramer *et al.* 2015), H4K20me1-mintbody was predominantly localized to chromosomes assumed to be the Xs and the artificial chromosome carrying *her-1* promoter sequences, compared to a broad and uniform expression of general chromatin protein H2B (Figure S3). In contrast, H4K20me1-mintbody and mTurquoise2::LacI did not co-localize in the nuclei of neurons with the artificial chromosome carrying only LacO repeats (Figure 1C). To quantify the co-localization of H4K20me1-mintbody and mTurquoise2::LacI, we measured the frequency of their co-localization in PLM neurons of each population. If fluorescence from the H4K20me1-mintbody and mTurquoise2::LacI appeared to have the same morphology and location, the spots were considered to be co-localized. The frequency of co-localization in worms carrying both LacO repeats and *her-1* promoter sequences on the array was significantly higher than that of worms carrying only LacO repeats (Figure 1E). These results indicate that localization of the H4K20me1-mintbody on the artificial chromosome was dependent on *her-1* promoter sequences. To rule out the possibility that any promoter sequences on the artificial chromosome receive H4K20me1 deposition, we tested the *abts-1.b* promoter, which is active in PLM neurons. No co-localization of the H4K20me1-mintbody and mTurquoise2::LacI was observed in worms with the artificial chromosome carrying LacO repeats and *abts-1.b* promoter sequences (Figure 1D, E). These observations are consistent with recognition of *her-1* promoter sequence-specific H4K20 mono-methylation on the artificial chromosome by the H4K20me1-mintbody.

We also calculated PCC values, a widely accepted statistical measure used to quantify co-localization (Dunn *et al.* 2011; McDonald and Dunn 2013). Worms with artificial chromosomes carrying *her-1* promoter sequences had a mean PCC of 0.31, which was significantly higher than that of worms with artificial chromosomes carrying only LacO or *abts-1.b* promoter sequences, -0.03 and 0.06, respectively (Figure 1F). Since positive PCC values indicate co-localization and a PCC value of zero suggests no association, these results provided convincing evidence for H4K20me1 modification of the artificial chromosomes carrying *her-1* promoter sequences.

**Figure 2:**
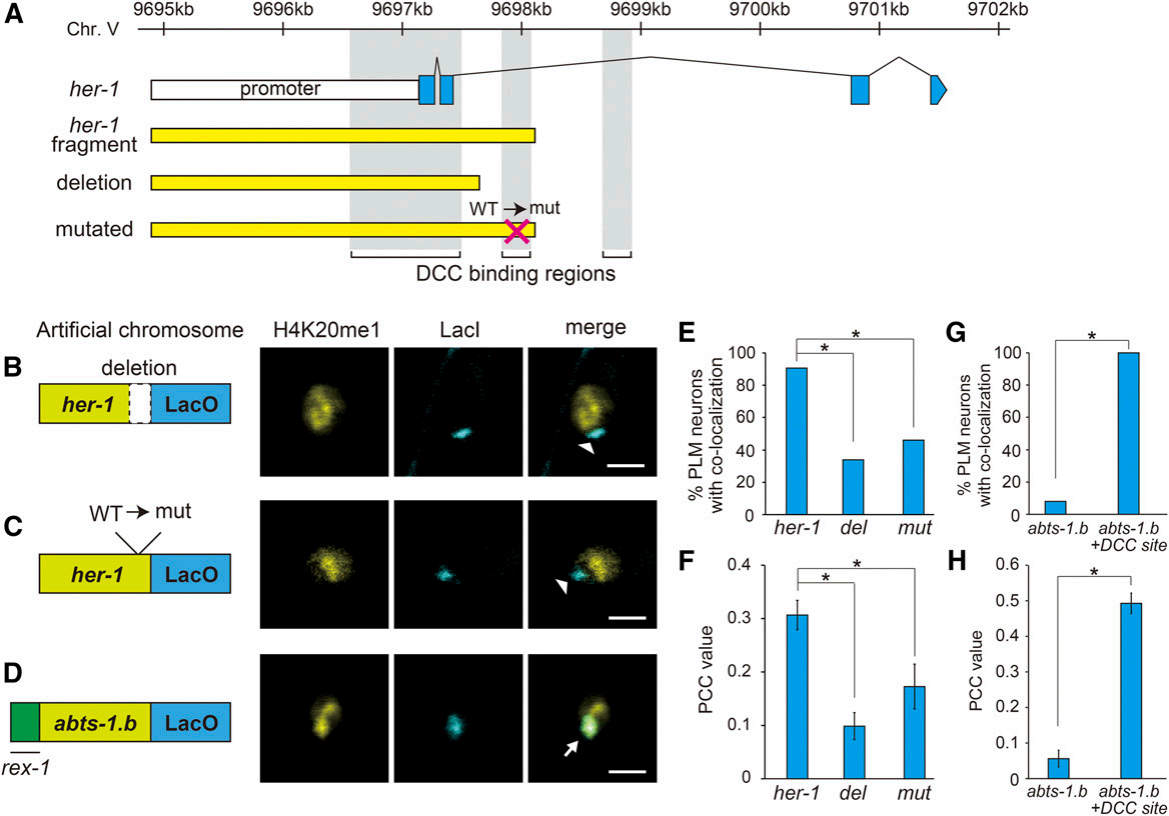
*her-1* sequence-specific H4K20me1 deposition on the artificial chromosome is dependent on DCC binding sites. (A) Structure of the *her-1* gene and position of DCC binding motifs. Blue boxes indicate *her-1* gene exons. Yellow boxes indicate the fragments used in each artificial chromosome. Vertical gray boxes indicate DCC binding regions. (B)–(D) Localization of the H4K20me1-mintbody and the artificial chromosomes carrying methylation-target and LacO sequences. (B) Short *her-1* sequences lacking one DCC binding site. (C) *her-1* sequences with a mutated DCC binding site. (D) *abts-1.b* promoter sequences with an ectopic DCC binding site (*rex-1*). All images are of the nuclei of PLM neurons at the L4 larval stage. Artificial chromosomes were visualized by the binding of mTurquoise2::LacI. Arrows indicate co-localization of the H4K20me1-mintbody and the artificial chromosome. Arrowheads indicate exclusion of H4K20me1-mintbody from the artificial chromosome. White scale bars, 2 µm. (E) Quantitative evaluation of the co-localization of H4K20me1-mintbody and artificial chromosomes carrying the mutant *her-1* and LacO sequences. *del* indicates the *her-1* sequence lacking one DCC binding site (B), and *mut* indicates the mutated *her-1* sequence (C). *P < 0.01. (F) Mean PCC values for the co-localization of H4K20me1-mintbody and artificial chromosomes corresponding to (E). *P < 0.01. (G) Quantitative evaluation of the co-localization of the H4K20me1-mintbody and artificial chromosomes carrying the *abts-1.b* promoter sequences with an ectopic DCC binding site (*rex-1*) and LacO sequences. *P < 0.01. (H) Mean PCC values for the co-localization of H4K20me1-mintbody and artificial chromosomes corresponding to (G). *P < 0.01.

To further validate the mintbody-LacO/LacI visualization method, we next investigated the DNA sequences important for H4K20me1 deposition on the artificial chromosomes. The *her-1* sequences used in this approach contained at least two known DCC binding sites (Figure 2A), which are critical for H4K20me1 deposition (Chu *et al.* 2002; Ercan *et al.* 2007). Using short *her-1* sequences, lacking one DCC binding site in the intron, the frequency of co-localization and mean PCC value were significantly reduced relative to those of the intact *her-1* sequence (Figure 2B, E, F). Similarly, mutations in the same DCC binding site also significantly reduced the frequency of co-localization and mean PCC value (Figure 2C, E, F). Next, we investigated whether the presence of a DCC binding site is sufficient for H4K20me1 deposition on the artificial chromosome and whether our system can reflect this modification. Previous reports have demonstrated that the *rex-1* sequence, a 33 bp DNA sequence for DCC binding, is present on the X chromosome, and that ectopic insertion of the *rex-1* sequence is sufficient for H4K20me1 deposition (Albritton *et al.* 2017; Brejc *et al.* 2017). As expected, the addition of *rex-1* to *abts-1.b* promoter sequences dramatically increased the frequency of co-localization of H4K20me1-mintbody and mTurquoise2::LacI in PLM neurons and the corresponding mean PCC value (Figure 2D, G, H). Interestingly, this similar co-localization was observed even if the extrachromosomal array (artificial chromosome) was integrated in the genome (Figure S4), suggesting that this method can detect the change of methylation state on the target sequence both in extrachromosomes and in the genome. These mechanism-based observations strongly suggest that our visualization method can detect H4K20me1 on DNA sequences from a specific gene locus in living worms.

**Figure 3:**
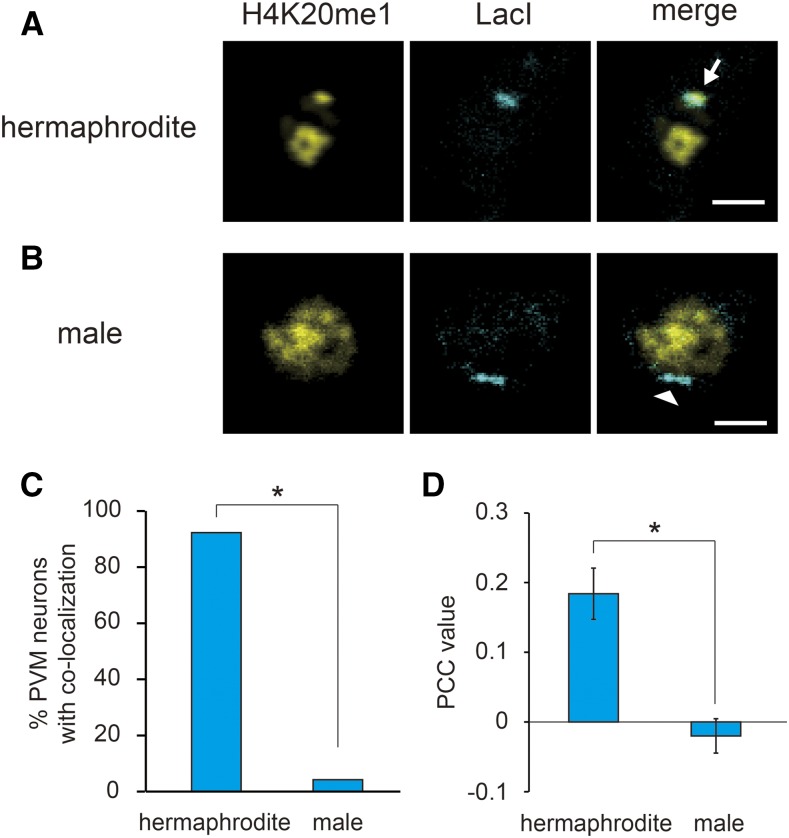
The mintbody-LacO/LacI system can reflect the sex-dependent alterations of H4K20me1 deposition on the *her-1* gene locus. (A)–(B) Localization of the H4K20me1-mintbody and the artificial chromosome carrying *her-1* and LacO sequences in PVM neurons of *him-5(e1490)* hermaphrodite (A) or male (B) worms. All images are at the L4 larval stage. Arrow indicates co-localization of H4K20me1-mintbody and the artificial chromosome, and arrowhead indicates exclusion of H4K20me1-mintbody from the artificial chromosome. White scale bars, 2 µm. (C) Quantitative evaluation of the co-localization of H4K20me1-mintbody and artificial chromosomes in hermaphrodite or male worms. *P < 0.01. (D) Mean PCC values of the co-localization of H4K20me1-mintbody and artificial chromosomes corresponding to (C). *P < 0.01.

We next sought to address whether this visualization method using artificial chromosomes can be applied to monitor context-dependent and developmental changes of histone modifications in the genome. Although *her-1* undergoes DCC binding and is transcriptionally repressed in hermaphrodite worms, the locus is reported to lose DCC binding and be actively transcribed to promote male-specific development in male worms (Li *et al.* 1999; Chu *et al.* 2002; Yonker and Meyer 2003; Ercan *et al.* 2007; Kramer *et al.* 2015). Thus, to confirm the sex-dependent alterations of H4K20me1, we compared the H4K20me1 modification of the artificial chromosome between hermaphrodites and male worms by observing the nuclei of PVM neurons, a type of mechanosensory neuron that has similar anatomical features in hermaphrodites and males. Whereas the H4K20me1-mintbody and mTurquoise2::LacI co-localized in the PVM neurons of hermaphrodite worms, no such co-localization was detected in male worms (Figure 3A, B, C). Moreover, the mean PCC values indicated that the H4K20me1-mintbody only co-localized with mTurquoise2::LacI in hermaphrodite worms (Figure 3D). Co-localization was also absent from the other neurons of male worms. Furthermore, the H4K20me1-mintbody appeared to be uniformly distributed in the nuclei of male worm cells, because of the lack of H4K20me1 deposition on the X chromosome (Figure 3B). Lastly, we examined whether our mintbody-LacO/LacI system can detect temporal changes of H4K20me1 deposition on the artificial chromosome in living cells. Using animals carrying *rex-1* and LacO sequences, we observed dynamic changes of both the position and fluorescent strength of H4K20me1 enriched regions within the nucleus of PLM neurons at the L2 larval stage, while H4K20me1-mintbody was stably maintained to co-localize with mTurquoise2::LacI during this period (Figure S5). Since context-dependent and temporal changes of H4K20me1 has been already completed at an early stage of embryogenesis, dynamic alteration of co-localization was not observed during this period. However, these results suggest that our visualization method using H4K20me1-mintbody and artificial chromosomes has the potential to detect temporal changes of histone modifications. It is reasonable to consider that the patterns of histone modification on artificial chromosomes reflect those on endogenous chromosomes, since many previous studies have shown that artificial chromosomes, which are used extensively for gene expression analysis, receive developmental and context-dependent modulations (Cochella and Hobert 2012; Towbin *et al.* 2012).

In summary, we present a method for visualization of H4K20me1 at a single-cell and single-gene resolution in a living model animal. *C. elegans* epigenetic ChIP assays using whole animals have generated numerous important findings; however, single-cell analysis of histone modifications to evaluate short-term changes in histone modification at specific gene loci has proven problematic. In this study, we demonstrate that H4K20me1 on specific DNA sequences from the *her-1* locus can be visualized in many types of neuronal cells and intestinal cells. These results suggest that our mintbody-LacO/LacI system could be applied to detect the states of locus-specific H4K20me1 in any kind of cells. Indeed, mintbody has been used to track the dynamics of H4K20me1 on X chromosomes in various cells during embryonic development in *C. elegans* (Sato *et al.* 2016). Together with the completely mapped entire cell lineage of *C. elegans*, the mintbody-LacO/LacI system will allow elucidation of the dynamics and roles of H4K20me1 on specific gene loci in particular cells of interest during development. Furthermore, since a mintbody recognizing histone H3 lysine 9 acetylation has been reported (Sato *et al.* 2013), our mintbody-LacO/LacI system could be applied to investigation of other histone modifications. The further development of intracellular antibodies against various kinds of epigenetic modifications will increase the importance of our system as a real-time imaging tool for epigenetic modifications with single-gene level resolution.

In addition, we adopted two approaches to evaluate co-localization. Our mintbody-LacO/LacI system allows measurement of changes in levels of H4K20me1 deposition by comparative analyses, as indicated by both mean PCC values and the frequency of co-localization. Indeed, these indices clearly identified alteration of H4K20me1 deposition levels at the mutant *her-1* sequence lacking one DCC binding site. Interestingly, the frequency of co-localization in animals carrying the mutant *her-1* sequence on the array was reduced, compared with that of animals carrying the intact *her-1* sequence on the array, but was higher than that of animals carrying only LacO sequences. These data suggest the possibility of relative quantitation of sequence-dependent H4K20me1 alteration using this visualization system. Furthermore, objective evaluation of co-localization by PCC was almost equivalent to subjective judgement of co-localization, based on the shape and location of H4K20me1-mintbody and mTurquoise2::LacI generated fluorescence. Thus, our visualization method can be used as a unique tool both for mutant screening by rapid visual inspection and for analysis of the complex molecular mechanisms involved in H4K20me1 dynamics by accurate relative quantitation.

Observing real-time alterations of H4K20me1 deposition on the target sequence is a big challenge for epigenetic studies. Previous reports showed that H4K20me1 enrichment on X chromosomes is evident after around the 300-cell stage of embryogenesis (Vielle *et al.* 2012; Sato *et al.* 2016; Brejc *et al.* 2017). In the embryonic stage, we could also observe the dynamic changes of H4K20me1 enrichment within the nuclei of several cells including intestinal nuclei. However, the signal of mTurquoise2:: LacI driven by the *ges-1* promoter, which starts transcription from about the 50-cell stage, could not be detected, probably because of the slow maturation of mTurquoise2 (data not shown). We believe that our visualization system using mintbody could be applied to detect real-time alteration of H4K20me1 deposition in living animals. However, we need to select suitable cells and the timing in which the H4K20 methylation states are dramatically altered. In addition, proper selection of fluorescent proteins with faster fluorophore maturation or promoters which drive transcription from an earlier developmental timepoint will be required for live imaging of H4K20me1 dynamics during embryogenesis.

Alternatively, our method may be quite helpful for genetic analyses. While existing methodologies using commercially available antibodies, such as ChIP assays and immunocytochemistry, are time-consuming and expensive, our method could be used to determine the phenotype of large numbers of worms, such as for genetic screening, and to rapidly identify histone modification regulators by genetic analyses. These features of our method compensate for the disadvantages of the major methods currently used for epigenetics studies. However, our mintbody-LacO/LacI system also has some drawbacks. Mintbody-LacO/LacI system does not visualize histone modifications on endogenous loci. Furthermore, as the H4K20me1-mintbody is differentially expressed in each cell and each transgenic worm, it is difficult to evaluate absolute amount of sequence-dependent histone modifications. Therefore, combined with the nature of artificial chromosomes carrying multiple copies of DNA sequences from a specific locus, our system may overestimate H4K20me1 in excess of endogenous level. Indeed, a subset of DCC binding sites estimated by artificial chromosomes (32%) did not overlap with those estimated by ChIP assays (Albritton *et al.* 2017). Thus, we believe that a combination of ChIP assays and our visualization method will compensate each other and offer a powerful tool to elucidate regulators, dynamics, and significance of histone modifications during various biological phenomena.
